# A New Platelet-Aggregation-Inhibiting Factor Isolated from* Bothrops moojeni* Snake Venom

**DOI:** 10.1155/2017/4315832

**Published:** 2017-11-01

**Authors:** Bruna Barbosa de Sousa, Carla Cristine Neves Mamede, Mariana Santos Matias, Déborah Fernanda da Cunha Pereira, Mayara Ribeiro de Queiroz, Edigar Henrique Vaz Dias, Anielle Christine Almeida Silva, Noelio Oliveira Dantas, Júnia de Oliveira Costa, Fábio de Oliveira

**Affiliations:** ^1^Instituto de Genética e Bioquímica, Universidade Federal de Uberlândia, Uberlândia, MG, Brazil; ^2^Instituto Nacional de Ciência e Tecnologia em Nano-Biofarmacêutica (N-Biofar), Belo Horizonte, MG, Brazil; ^3^Instituto de Ciências Agrárias, Universidade Federal de Uberlândia, Monte Carmelo, MG, Brazil; ^4^Instituto de Física, Universidade Federal de Uberlândia, Uberlândia, MG, Brazil; ^5^Instituto Federal de Educação, Ciência e Tecnologia do Triângulo Mineiro, Campus Ituiutaba, Ituiutaba, MG, Brazil; ^6^Instituto de Ciências Biomédicas, Universidade Federal de Uberlândia, Uberlândia, MG, Brazil

## Abstract

This work reports the purification and functional characterization of BmooPAi, a platelet-aggregation-inhibiting factor from* Bothrops moojeni* snake venom. The toxin was purified by a combination of three chromatographic steps (ion-exchange on DEAE-Sephacel, molecular exclusion on Sephadex G-75, and affinity chromatography on HiTrap™ Heparin HP). BmooPAi was found to be a single-chain protein with an apparent molecular mass of 32 kDa on 14% SDS-PAGE, under reducing conditions. Sequencing of BmooPAi by Edman degradation revealed the amino acid sequence LGPDIVPPNELLEVM. The toxin was devoid of proteolytic, haemorrhagic, defibrinating, or coagulant activities and induced no significant oedema or hyperalgesia. BmooPAi showed a rather specific inhibitory effect on ristocetin-induced platelet aggregation in human platelet-rich plasma, whereas it had little or no effect on platelet aggregation induced by collagen and adenosine diphosphate. The results presented in this work suggest that BmooPAi is a toxin comprised of disintegrin-like and cysteine-rich domains, originating from autolysis/proteolysis of PIII SVMPs from* B. moojeni* snake venom. This toxin may be of medical interest because it is a platelet aggregation inhibitor, which could potentially be developed as a novel therapeutic agent to prevent and/or treat patients with thrombotic disorders.

## 1. Introduction

Snake venoms comprise pharmacologically active proteins and peptides, both enzymatic and nonenzymatic, such as phospholipases A_2_, metalloproteinases, serine proteinases, nucleotidases, L-amino acid oxidase, disintegrins, and C-type lectins [1–4]. Several snake venom metalloproteinases (SVMPs) have been isolated and characterized by their biological activities. These enzymes play a key role in the prominent local tissue damage and systemic alterations caused by snake venom. SVMPs induce haemorrhage, myonecrosis, skin damage, inflammation, and degradation of extracellular matrix components. In addition, some SVMPs affect platelet function, while others degrade blood clotting factors, potentiating the haemorrhagic effect [4–8].

SVMPs comprise a group of zinc-dependent enzymes of varying molecular mass, widely distributed in Viperidae venoms. They are synthesized as multidomain precursors and stored in the venom gland as inactive zymogens [7, 9–11]. SVMPs are classified into three major classes, PI, PII, and PIII, according to their size (molecular mass) and domain organization. PI SVMPs include small metalloproteinases with only the metalloproteinase domain. PII SVMPs comprise medium-size proteinases composed of one metalloproteinase and one disintegrin domain. PIII SVMPs have an additional cysteine-rich domain following the disintegrin-like domain and, in some cases, a lectin-like domain. PII and PIII SVMPs are divided into several subclasses based on proteolytic processing. PII SVMPs can be processed into a metalloproteinase domain and a nonenzymatic disintegrin, and PIII SVMPs can also be degraded, releasing a stable fragment which corresponds to the disintegrin-like and cysteine-rich domains (dis-cys domain) [12, 13].

Several studies have investigated SVMPs as platelet aggregation inhibitors due to their specificity to platelet integrins [4, 7, 10, 14–18]. The disintegrin domain usually contains the RGD (Arg-Gly-Asp) or KGD (Lys-Gly-Asp) motifs in its inhibitory loop, which binds with a high degree of selectivity to the *α*_IIb_*β*_3_ platelet integrin (also known as GPIIb/IIIa), blocking the last phase of platelet aggregation and clot formation [18, 19]. Therefore, SVMPs are considered potent platelet aggregation inhibitors [7]. Unlike the disintegrin domain, whose integrin-binding motif is well characterized, the dis-cys domain possesses the XXCD (X-X-Cys-Asp) motif, instead of the usual RGD, and its targets are not yet fully elucidated. The extant literature indicates that the biological activities attributed to this domain result from its ability to block the interaction of platelets with collagen [19].

In the present study, we describe the isolation and functional characterization of BmooPAi, a toxin comprised of the dis-cys domain, originating from autolysis/proteolysis of PIII SVMPs from* Bothrops moojeni* snake venom, which showed an inhibitory effect on platelet aggregation.

## 2. Materials and Methods

### 2.1. Material

Desiccated* B. moojeni* venom was purchased from Bioagents Serpentarium (Batatais, SP, Brazil). Acrylamide, ammonium bicarbonate, ammonium persulphate, azocasein, bromophenol blue, ethylenediaminetetracetic acid (EDTA), bovine fibrinogen, glycine, *β*-mercaptoethanol, *N*,*N*′-methylene-*bis*-acrylamide, sodium dodecyl sulphate (SDS), *N*,*N*,*N*′,*N*′-tetramethylethylenediamine (TEMED), and Tris were purchased from Sigma Chemical Co. (St. Louis, MO, USA). Molecular weight markers for electrophoresis and all chromatographic media (DEAE-Sephacel, Sephadex G-75, HiTrap Heparin HP and C2/C18 columns) were purchased from GE Healthcare Technologies (Uppsala, Sweden). All the agonists used in the platelet aggregation assays (collagen from equine tendon, adenosine diphosphate (ADP), and ristocetin) were purchased from Helena Laboratories (Beaumont, Texas, USA). All other reagents used were of analytical grade.

### 2.2. Animals

Male Swiss mice (20–25 g) and male Wistar rats (200–250 g) were provided by the Centro de Bioterismo e Experimentação Animal (CEBEA) at the Federal University of Uberlândia (Uberlândia-MG, Brazil). The animals were maintained under conditions of controlled temperature (22 ± 2°C) and light/dark cycle (12 hours) with free access to food and water. The experimental protocol was approved by the Committee for Ethics in Animal Experimentation of the Federal University of Uberlândia (CEUA/UFU), Minas Gerais, Brazil (Protocol number 108/12).

### 2.3. Human Blood

The experiments were performed in accordance with current guidelines for human research, established by the Committee for Ethics in Human Research of the Federal University of Uberlândia, Minas Gerais, Brazil (CEP/UFU, Protocol number 1.627.982/2016). Blood was obtained by blood donation from 10 individuals who were invited to participate in the research as volunteer donors. The criteria for the selection of donor volunteers included no signs or symptoms of disease, malnutrition, or dehydration; age between 18 and 65 years; weight more than 50 kg; no use of any medication that interferes with haemostasis; no use of illicit drugs; no alcohol consumption in the last 24 hours preceding the experiment; and no haemostasis disorders.

### 2.4. Isolation of BmooPAi

Protein isolation was carried out in three steps. The crude venom of* B. moojeni* (200 mg) was dissolved in 2.0 mL of 0.05 M ammonium bicarbonate buffer (pH 7.8) and clarified by centrifugation at 10,000 ×g for 10 min. The supernatant was applied to a DEAE-Sephacel column (2.5 × 20 cm) previously equilibrated with 0.05 M ammonium bicarbonate buffer (pH 7.8). Chromatography was carried out at a flow rate of 20 mL/h, with a linear concentration gradient of the same buffer (0.05–0.6 M), and fractions of 3.0 mL/tube were collected. All peaks were monitored by measuring absorbance at 280 nm on a spectrophotometer (BioSpec-Mini; Shimadzu Biotech, Japan). The seventh peak, designated D7, was pooled, lyophilised, and applied to a Sephadex G-75 column (1.0 × 100 cm) previously equilibrated with 0.05 M ammonium bicarbonate buffer (pH 7.8). The samples were eluted from this column with the same buffer, at a flow rate of 20 mL/h, and fractions of 3.0 mL/tube were collected. The second fraction, designated D7S2, was pooled, lyophilised, and submitted to the third step of separation using a HiTrap Heparin HP column (5 × 1 mL) in an ÄKTApurifier™ HPLC system, previously equilibrated with 20 mM Tris-HCl buffer (pH 7.0) containing 5 mM calcium chloride. The samples were eluted with an increasing concentration gradient of 20 mM Tris-HCl buffer (pH 7.0) containing 2.0 M sodium chloride. Elution was carried out at a flow rate of 30 mL/h; fractions of 1.0 mL/tube were collected and the absorbance was read at 280 nm. Isolated BmooPAi was concentrated in the major peak. To evaluate the degree of purity, isolated BmooPAi was passed through a reverse-phase C2/C18 column (4.6 × 100 mm) using the ÄKTApurifier HPLC system. The column was equilibrated with solvent A (0.1% trifluoroacetic acid) and eluted with a linear concentration gradient from 0 to 100% of solvent B (70% acetonitrile, 0.1% trifluoroacetic acid) at a flow rate of 0.3 mL/min. Absorbance was monitored at 280 nm.

### 2.5. Protein Analysis

The protein concentration was determined using the method established by Bradford [20]. Determination of the protein concentration was performed in triplicate and the absorbance was measured at 595 nm. The protein concentration (mg/*μ*L) was determined from linear regression calculations based on the values obtained from the standard curve. Polyacrylamide gel electrophoresis in the presence of sodium dodecyl sulphate (SDS-PAGE) was performed using 14% (w/v) gels. Electrophoresis was carried out at 20 mA/gel in Tris-glycine buffer (pH 8.3) containing 0.01% SDS. The molecular mass standard proteins used included phosphorylase b (97 kDa), bovine serum albumin (66 kDa), ovalbumin (45 kDa), carbonic anhydrase (30 kDa), soybean trypsin inhibitor (20.1 kDa), and *α*-lactalbumin (14.4 kDa). Gels were stained with Coomassie blue R-250, 0.2% (w/v). The relative molecular mass of BmooPAi was estimated using Kodak 1D image analysis software.

### 2.6. N-Terminal Sequencing

The sequencing was performed in a membrane obtained from the purified protein in solution. The liquid sample was loaded onto an Applied Biosystems Prosorb™ device. The protocol for sample preparation using this device was followed, and the Prosorb membrane was loaded onto the instrument for sequence analysis.

### 2.7. Proteolytic Activity upon Fibrinogen

Fibrinogenolytic activity was assayed as previously described [21], with brief modifications. Samples containing 25 *μ*L of bovine fibrinogen (3 mg/mL saline) were incubated with 20 *μ*g of BmooPAi for 120 min at 37°C. The reaction was stopped by the addition of a denaturing buffer containing 10% (v/v) glycerol, 10% (v/v) *β*-mercaptoethanol, 0.2% (w/v) SDS, and 0.001% (w/v) bromophenol blue (pH 6.8). Reaction products were analysed using 14% (w/v) SDS-PAGE.

### 2.8. Proteolytic Activity upon Azocasein

Samples of 20 *μ*g BmooPAi were supplemented with 200 *μ*L of saline and incubated for 60 min with 800 *μ*L of azocasein solution (1 mg/mL). The reaction was stopped by the addition of 200 *μ*L of 15% trichloroacetic acid to precipitate the undegraded azocasein. The mixture was left standing for 20 min before centrifugation at 10,000 ×g for 10 min. The proteolytic activity was estimated by reading the absorbance of the clear supernatant at 405 nm. One unit of azocaseinolytic activity was defined as the amount of enzyme that produced an absorbance increase of 0.01 units.

### 2.9. Haemorrhagic Activity

Haemorrhagic activity was determined as previously described [22], with some modifications. Groups of three male Swiss mice were administered a dorsal skin subcutaneous injection of 50 *μ*g BmooPAi diluted in 25 *μ*L of saline. After three hours, animals were euthanized by an overdose of ketamine/xylazine, the skin was removed and the diameter of the haemorrhagic spot was measured on the inner surface. Control animals received the same volume of sterile saline.

### 2.10. Defibrinating Activity

Defibrinating activity was carried out as previously described [23], with slight modifications. Groups of three male Swiss mice were intraperitoneally injected with 50 *μ*g BmooPAi (50 *μ*g/100 *μ*L saline). Control animals received the same volume of sterile saline. After one hour, mice were euthanized by an overdose of ketamine/xylazine and bled by cardiac puncture. Whole blood was placed in tubes and kept at 25–30°C. Activity was determined by measuring the time to the onset of blood clotting.

### 2.11. Coagulant Activity

Coagulant activity was assayed using bovine plasma. The plasma samples were mixed with 3.8% sodium citrate (9 : 1, v/v) and centrifuged at 2,500 ×g for 15 min to obtain platelet-rich plasma (PRP). BmooPAi (25 *μ*g/25 *μ*L), the same volume of saline (negative control), or 0.2 mol/L calcium chloride (positive control) was added to 200 *μ*L of citrated bovine PRP at 37°C. Clotting activity was determined by measuring the time to fibrin clot onset by a coagulometer (CLO Timer).

### 2.12. Evaluation of Paw Oedema Formation

Groups of three male Wistar rats received an intraplantar injection of BmooPAi (50 *μ*g/100 *μ*L saline) into the subplantar region of one hind paw. An equal volume of sterile saline was injected into the contralateral paw. The volume of each paw was measured using a plethysmometer (model 7140; Ugo Basile, Italy) before and 1, 2, 3, 4, 5, 6, and 24 h after the injection. Results were calculated as the difference between each paw and expressed as the percentage increase in paw volume relative to the initial volume.

### 2.13. Evaluation of Hyperalgesia

Hyperalgesia was measured according to the paw pressure test, as previously described [24], with slight modifications. Groups of three male Wistar rats received an intraplantar injection of BmooPAi (50 *μ*g/100 *μ*L saline) into the right hind paw. An equal volume of sterile saline was injected into the left hind paw for control. The weight in grams (g) required to elicit a nociceptive response, that is, paw flexion, was determined as the nociceptive threshold. A cut-off value of 300 g was used to prevent damage to the paws. The nociceptive threshold was measured before and 1, 2, 3, 4, 5, 6, and 24 h after BmooPAi injection using a Randall-Selitto apparatus (EFF-440 model; Insight, Brazil). Results were calculated as the difference between each paw and expressed as the percentage decrease in nociceptive threshold relative to the initial threshold. To reduce stress, the rats were habituated to the apparatus one day before the experiment.

### 2.14. Platelet Aggregation Assay

Platelet aggregation assays were performed as previously described [25] in human PRP and measured using the automatic Aggregometer 4 channels (AggRAM™ version 1.1; Helena Laboratories, USA). Human blood collected in sodium citrate was centrifuged at 100 ×g for 12 min at room temperature to obtain PRP. Platelet-poor plasma (PPP) was obtained from the residue by centrifugation of citrated blood at 1,000 ×g for 15 min. Assays were carried out using 200 *μ*L of PRP maintained at 37°C under continuous stirring, in siliconized glass cuvettes. Aggregation was triggered with collagen (10 *μ*g/mL), ADP (20 *μ*M), or ristocetin (1.5 mg/mL) immediately after adding BmooPAi (10, 25 and 50 *μ*g) to human PRP. Finally, 100% aggregation was expressed as the percentage absorbance relative to PPP aggregation. Control experiments were performed using only platelet agonists. All experiments were carried out in triplicate.

## 3. Results and Discussion

In recent years, a number of snake venom proteins that interfere with platelet function have been identified. Most of these proteins belong to the SVMP family, and their effects are probably due to the presence of dis-cys domains which interact with specific protein molecules on platelet membrane surfaces [25–29]. In this work, we report the purification and functional characterization of BmooPAi, a platelet-aggregation-inhibiting factor, comprised of dis-cys domains processed from PIII SVMPs from* B. moojeni* snake venom.

**Figure 1:**
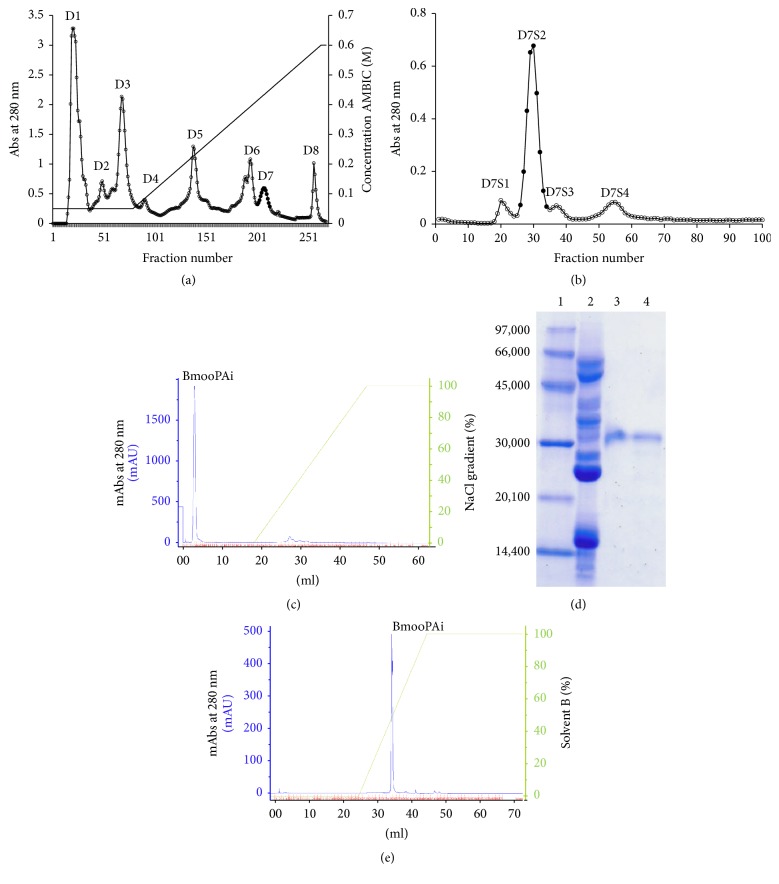
Purification of BmooPAi from* B. moojeni* snake venom. (a) Separation on DEAE-Sephacel ion-exchange chromatography: crude venom (200 mg) was applied to the column (2.5 × 20 cm) and elution was carried out at a flow rate of 20 mL/h with ammonium bicarbonate (Ambic) buffer gradients, pH 7.8, from 0.05 M to 0.6 M. Fractions of 3.0 mL/tube were collected and the absorbance was read at 280 nm. (b) Separation on Sephadex G-75 molecular exclusion chromatography: fraction D7 was applied to the column (1.0 × 100 cm) and elution with 0.05 M ammonium bicarbonate was achieved at a flow rate of 20 mL/h. Fractions of 3.0 mL/tube were collected and the absorbance was read at 280 nm. (c) Separation by affinity chromatography on a HiTrap Heparin HP column using the ÄKTApurifier HPLC system: fraction D7S2 was applied to the column (5 × 1 mL), previously equilibrated with 20 mM Tris-HCl buffer (pH 7.0) containing 5 mM calcium chloride. The samples were eluted with an increasing concentration gradient of 20 mM Tris-HCl buffer (pH 7.0) containing 2.0 M sodium chloride, and the absorbance of the fractions was monitored at 280 nm. Fractions of 1.0 mL/tube were collected at a flow rate of 30 mL/h. (d) SDS-PAGE in 14% (w/v) polyacrylamide, Tris-glycine buffer, pH 8.3, and 20 mA. Lanes: 1, standard proteins; 2, reduced crude venom of* B. moojeni*; 3, reduced BmooPAi; 4, nonreduced BmooPAi. The gel was stained with Coomassie blue R-250. (e) Reverse-phase HPLC on a C2C18 column (4.6 × 100 mm) equilibrated with 0.1% trifluoroacetic acid (TFA) and eluted with a linear concentration gradient from 0 to 100% of solution B (70% acetonitrile in 0.1% TFA).

The toxin was isolated using a three-step procedure, including ion-exchange, gel filtration, and affinity chromatographies. Other toxins have been purified using similar procedures, including BmooFIBMP-I [30] and BmooMP*α*-1 [31], both metalloproteinases purified from* B. moojeni* snake venom.* B. moojeni* crude venom (200 mg) was applied to ion-exchange chromatography on a DEAE-Sephacel column, which produced eight main protein peaks, designated D1 to D8 (Figure 1(a)). The D7 fraction represented around 5.5% (w/w) of the crude venom; it was submitted to gel filtration chromatography on a Sephadex G-75 column, resulting in four peaks, designated D7S1 to D7S4 (Figure 1(b)). The D7S2 fraction (~3.3%) was further fractionated over an affinity chromatography HiTrap Heparin HP column using the ÄKTApurifier HPLC system (Figure 1(c)). The nonadsorbed fraction (~1.7%) was able to inhibit platelet aggregation and it was designated BmooPAi (platelet aggregation inhibitor from* B. moojeni*). This fraction was devoid of proteolytic, haemorrhagic, defibrinating, or coagulant activities. In addition, BmooPAi did not cause hyperalgesia and oedematogenic responses in the hind paw when rats received a 50 *μ*g intraplantar injection of the toxin. Thus, BmooPAi does not contribute to the local effects caused by* B. moojeni* envenomation.

The homogeneity of this sample was demonstrated by SDS-PAGE, showing a single protein band around 32 kDa in the presence or absence of the reducing agent *β*-mercaptoethanol (Figure 1(d)). The degree of purity of the isolated BmooPAi was confirmed by reverse-phase HPLC chromatography on a C2/C18 column, revealing a unique major peak (Figure 1(e)).

**Figure 2:**
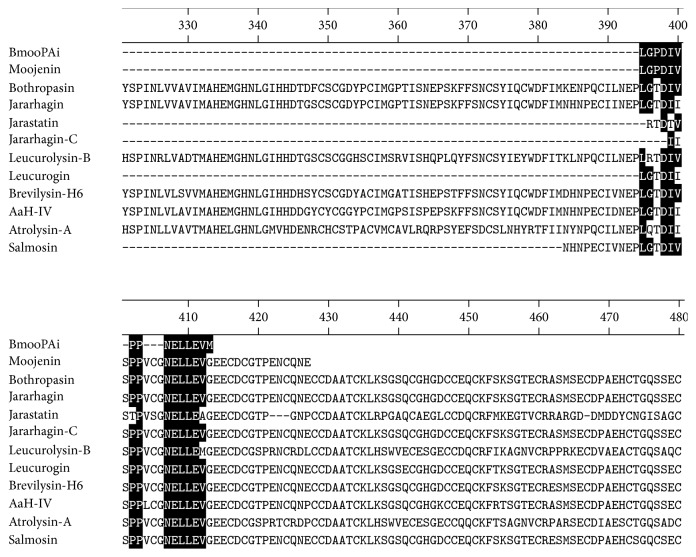
Sequence alignment of BmooPAi and other metalloproteinases/dis-cys proteins: Moojenin (GI: P0DKR0.1), Bothropasin (GI: 209870468), Jararhagin (GI: P30431.1), Jarastatin (GI: Q0NZX5.1), Jararhagin-C (GI: P30431.1), Leucurolysin-B (GI: P86092.1), Leucurogin (GI: P0DJ87.1), Brevilysin-H6 (GI: P0C7B0.2), AaH-IV (GI: 255917952), Atrolysin-A (GI: Q92043.1), and Salmosin (GI: O93518.1). The nonconserved residues are shown in black frames. The alignment and figure were generated by the MegAlign program from Lasergene (DNAStar Inc., Madison, WI, USA).

BmooPAi was subjected to N-terminal sequencing by Edman degradation. The first 15 amino acid residues from N-terminal sequencing were determined to be LGPDIVPPNELLEVM and were submitted to BLAST. The primary sequence of BmooPAi shared similarity with the middle of some PIII SVMPs, skipping the catalytic domain, and with some disintegrin or disintegrin-like proteins. Moojenin (GI: P0DKR0.1), Bothropasin (GI: 209870468), Jararhagin (GI: P30431.1), Leucurolysin-B (GI: P86092.1), Brevilysin-H6 (GI: P0C7B0.2), Atrolysin-A (GI: Q92043.1), and AaH-IV (GI: 255917952) are PIII SVMPs that showed homology with BmooPAi; Jarastatin (GI: Q0NZX5.1), Jararhagin-C (GI: P30431.1), Leucurogin (GI: P0DJ87.1), and Salmosin (GI: O93518.1) are disintegrin or disintegrin-like proteins homologous to BmooPAi (Figure 2).

PIII SVMPs comprise high molecular mass proteinases composed of a dis-cys domain following the proteinase domain. Some PIII SVMPs have an additional lectin-like domain linked to the others through a disulphide bond [12, 13]. SVMPs play an important role in haemostatic disorders. They are the primary factors responsible for haemorrhage [32]. PIII SVMPs are more able to induce haemorrhage than PI and PII classes due to the presence of the additional dis-cys domain which contributes to the proteolytic specificity of these enzymes, directing them to bind to critical components of the microvasculature [28, 33]. Subclass PIIIb SVMPs can undergo proteolysis/autolysis during secretion or under nonphysiological conditions* in vitro*, such as alkaline pH, low calcium, or the presence of reducing agents, generating intact biologically active polypeptides of ~30 kDa, which correspond to the dis-cys domain [13, 34].

Although BmooPAi showed high homology with many PIII SVMPs, it showed no haemorrhagic activity. These data suggest that this toxin originated from the proteolysis/autolysis of a PIII SVMP and comprises only the noncatalytic dis-cys domain. For this reason, BmooPAi was not able to induce haemorrhage due to the absence of the proteinase domain, preventing proteolysis of the capillary components.

Some PIII SVMPs homologous to BmooPAi, such as Bothropasin [35], Brevilysin-H6 [36], Leucurolysin-B [37], AaH-IV [38], and Jararhagin [39], can be cleaved through autoproteolytic events, releasing the dis-cys domain. According to the characteristics of BmooPAi, we suggest that this toxin is similar to Jararhagin-C (28 kDa) [40] and Acucetin (30 kDa) [41]. Both toxins are composed of the dis-cys domain with a molecular mass of approximately 30 kDa released through autoproteolysis of Jararhagin from* B. jararaca* [39] and AaH-IV from* Agkistrodon acutus* [38]. Similar to BmooPAi, Jararhagin-C and Acucetin do not induce haemorrhage. For this reason, we suggest that BmooPAi originates from the class of PIII SVMPs, as proposed by Fox and Serrano [13], in which the dis-cys domain has been processed from the proteinase domain.

**Figure 3:**
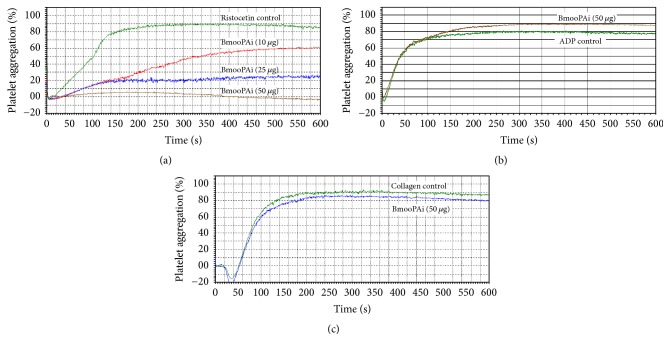
Effect of BmooPAi from* B. moojeni* venom on (a) ristocetin-, (b) ADP-, and (c) collagen-induced platelet aggregation. Aggregation was triggered with the agonists immediately after adding the indicated doses of BmooPAi to citrated human PRP at 37°C. Platelet aggregation was recorded for 10 min in an AggRAM platelet aggregation system with four-channel laser optics (Helena Laboratories, EUA). Results are expressed as an increase in light transmission, where PPP represents the maximum response (100%). Control experiments were performed in the absence of BmooPAi.

The extant literature shows that several PIII SVMPs and processed dis-cys domains are able to interfere with platelet aggregation, including Jararhagin [39], Jararhagin-C [40], Alternagin-C [42], Leucurogin [43], and Leberagin-C [44]. Their targets on platelet membranes include the receptors GPIb-IX-V, *α*_IIb_*β*_3_ integrin (GPIIb-IIIa), *α*_2_*β*_1_ integrin, and GPVI and also their ligands, von Willebrand factor (vWF), fibrinogen, and/or collagen [4, 45]. In this work, BmooPAi was tested for its effect on platelet aggregation.  Figure 3(a) shows that BmooPAi inhibited platelet aggregation induced by ristocetin in a dose-dependent manner, although it had little or no effect on platelet aggregation induced by ADP (Figure 3(b)) and collagen (Figure 3(c)). Our results showed that 10 and 25 *μ*g of BmooPAi inhibited approximately 32% and 72% of ristocetin-induced platelet aggregation, respectively, whereas 50 *μ*g completely inhibited platelet aggregation induced by ristocetin. On the other hand, BmooPAi was not able to inhibit the platelet aggregation induced by ADP and collagen, even at high concentrations (50 *μ*g).

According to Coller and Gralnick [46], ristocetin is an antibiotic that induces vWF-GPIb binding* in vitro*, even at static conditions. This interaction plays a key role in the initial adhesion of platelets to the subendothelium, which in turn induces a complex cascade of signals which results in platelet aggregation [47]. Since the circulating native form of vWF does not bind to platelet GPIb under static conditions, the antibiotic ristocetin allows the reproduction of the platelet aggregation events* in vitro* [48]. The importance of vWF-GPIb binding has been demonstrated for the clinical subsidiary diagnosis of hemostatic disorders, such as Bernard-Soulier syndrome [49] and von Willebrand disease [50].

A number of haemostatically active proteins that inhibit ristocetin (or botrocetin, a snake venom protein that also promotes* in vitro* vWF-GPIb binding)-induced platelet aggregation have been isolated and characterized from the venom of several snake species [3, 15]. Echicetin [51], Agkicetin [52], Jararaca GPIb-BP [53], Tokaracetin [54], Crotalin [55], Jararhagin [56], Kaouthiagin [57], Acurhagin [58], Kistomin [59], and Mocarhagin [60] are some examples.

Echicetin, Agkicetin, Jararaca GPIb-BP, and Tokaracetin toxins belong to the C-type lectin family and are able to bind directly to GPIb, blocking the binding of vWF to GPIb [61]. Jararhagin, Kaouthiagin, Acurhagin, Kistomin, Mocarhagin, and Crotalin belong to the SVMP family and are able to inhibit ristocetin-induced platelet aggregation due to their catalytic effect on GPIb and/or vWF [15]. BmooPAi also interferes with the vWF-GPIb interaction, but since it has no catalytic effect, we suggest that BmooPAi acts as an antagonist of the GPIb receptor, as described in other works. It is probable that the presence of the dis-cys domain directs the toxin to the platelet receptor. Furthermore, as BmooPAi did not inhibit ADP- or collagen-induced platelet aggregation, we can rule out the possibility of activity toward *α*_2_*β*_1_, GPVI, P2Y1, and P2Y12 receptors. The initial findings from this work in progress provide impetus to further investigate the mechanism of action of BmooPAi on the platelet receptor.

## 4. Conclusions

In conclusion, we describe the procedures for isolation of BmooPAi (32 kDa), a toxin comprised of the dis-cys domain, originating from autolysis/proteolysis of PIII SVMPs from* B. moojeni* venom. BmooPAi may be of medical interest because it is a platelet aggregation inhibitor that interferes with the vWF-GPIb interaction. This toxin can be used to better understand the mechanisms concerning haemostasis and could potentially be used as a tool for the development of novel antithrombotic agents.
